# Supplementary material for: Targeted Selected Reaction Monitoring Verifies Histology Specific Peptide Signatures in Epithelial Ovarian Cancer
Source: Cancers (Basel). 2021 Nov 15;13(22):5713. doi: 10.3390/cancers13225713 (PMC8616310; doi:10.3390/cancers13225713)
Supplement: Supplementary file 1 [file cancers-13-05713-s001.zip › cancers-1408976-supplementary-figure-revised.pdf]

SF1a.

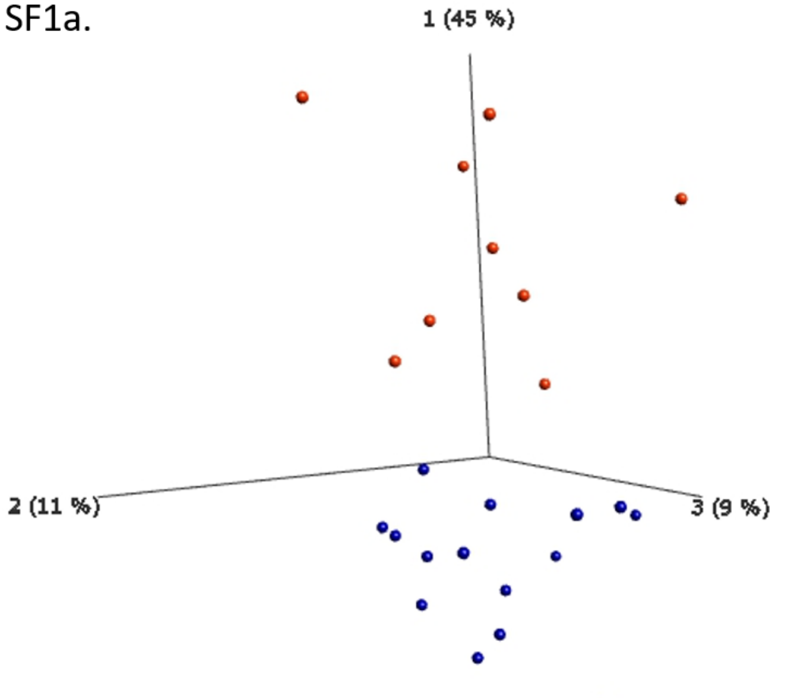
1 (45 %)
2 (11 %)
3 (9 %)

SF1b.

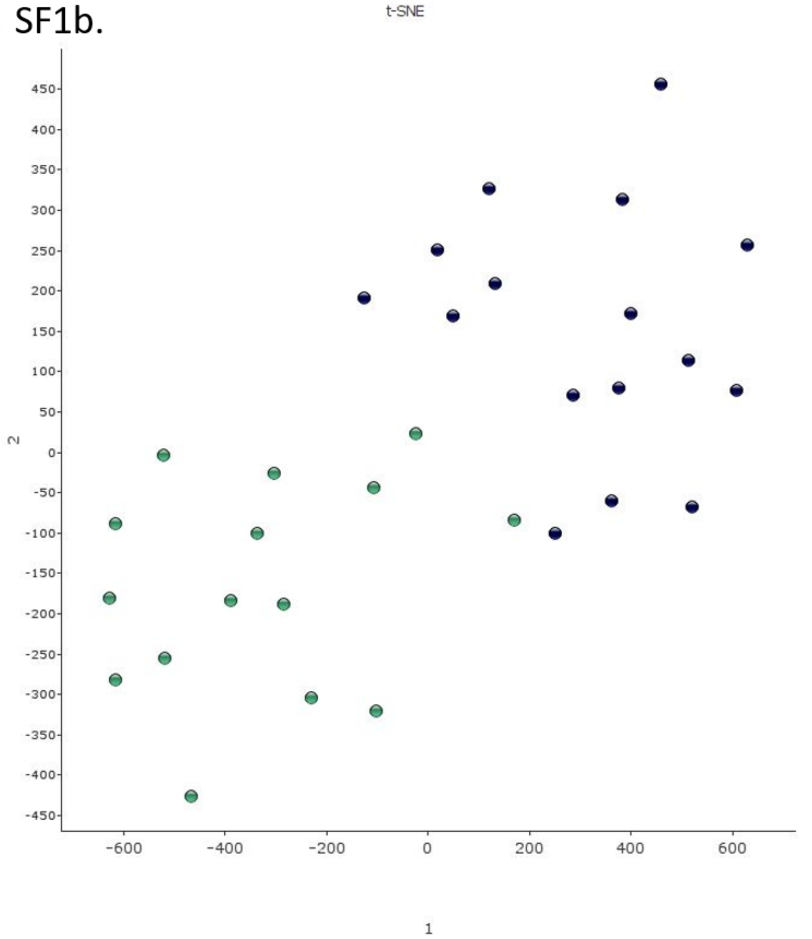
t-SNE
450
400
350
300
250
200
150
100
50
0
-50
-100
-150
-200
-250
-300
-350
-400
-450
2
-600
-400
-200
0
200
400
600
1

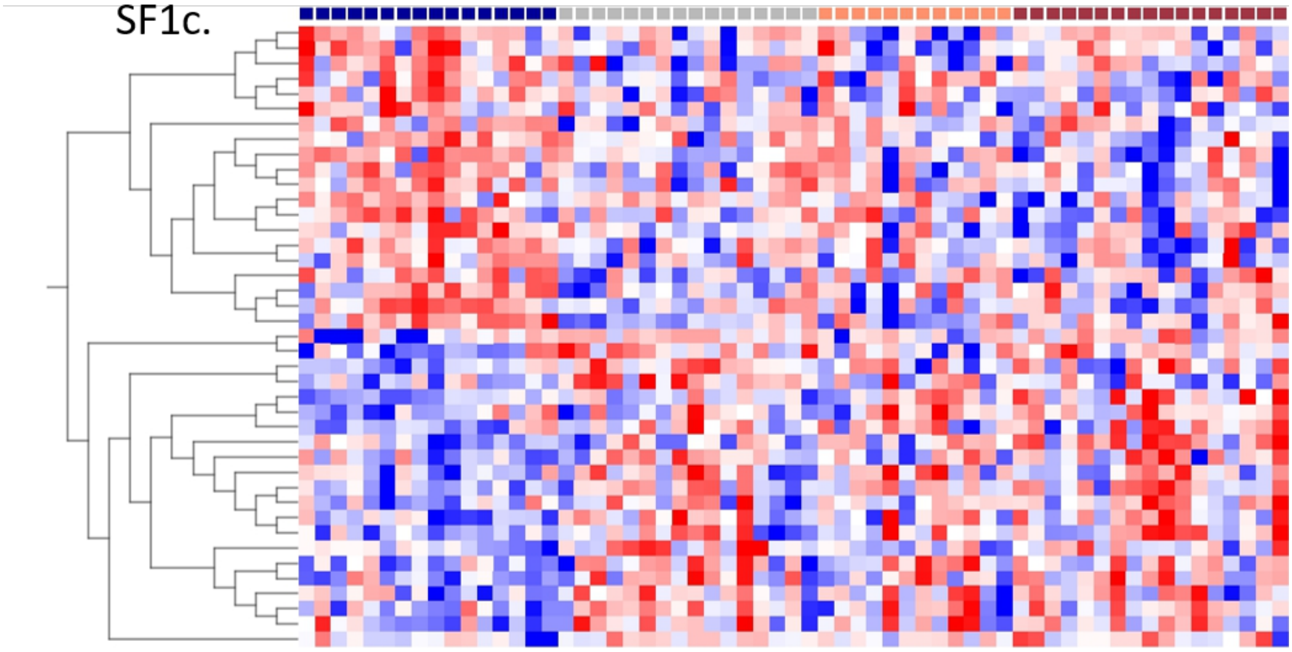
SF1c.

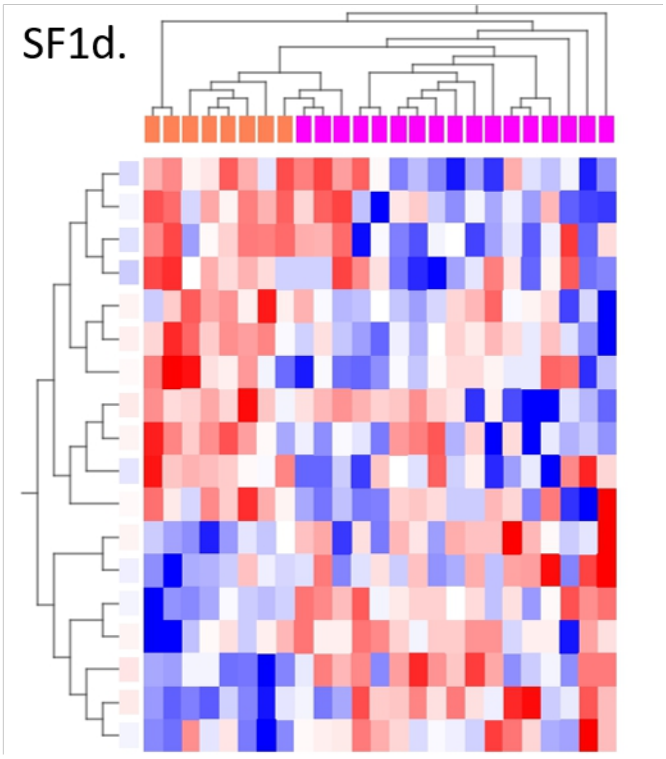
SF1d.

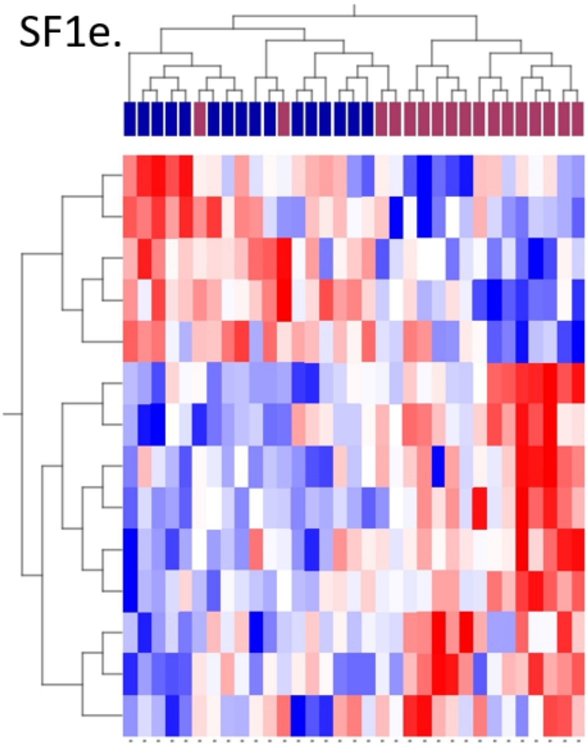

Supplementary Figure S1. Examples of plots from the initial data exploratory phase using Qlucore Omics tools **(a)** Principal component analysis of benign (blue) and mucinous (orange) samples, with a p-value filter for the two groups of p=0.01, q=0.07, resulting in 19 peptides **(b)** t-distributed Stochastic Neighbour Embedding (t-SNE) with benign tumors (blue) and low-grade Stage I ovarian cancer (green) with p=0.01, q=0.085, resulting in 19 peptides, **(c)** Hierarchical clustering of peptides, benign (blue) vs. malignant ovarian tumors, p=0.05 resulting in 41 peptides. Malignant cancers sorted according to group with low-grade (grey), high-grade stage I-II (orange) and HGSC stage III (bordeaux) **(d)** Hierarchical clustering of both samples and peptides of HGSC stage I-II vs. III resulting in 18 peptides at p=0.050 **(e)** Hierarchical clustering of both samples and peptides, benign tumors (blue) and HGSC stage III (bordeaux) with p<0.01, q=0.05, 14 peptides.
